# Empowering nurses: exploring self-managed organizations in Indian healthcare

**DOI:** 10.1186/s12912-023-01647-5

**Published:** 2023-12-15

**Authors:** Elham Malik, Shail Shankar

**Affiliations:** Department of Humanistic Studies, Indian Institute of Technology (BHU), Varanasi, Uttar Pradesh India

**Keywords:** Nursing experience, Self-managed organizations (SMOs), Homecare, Nursing practices in SMOs

## Abstract

**Background:**

Given India’s high patient load on the existing healthcare setup, as well as political, social, and organizational challenges, the nursing sector is facing various problems, therefore leading to substandard nursing experiences leading to poor patient care at the parallel healthcare setups, specifically homecare. This paper presents self-managed organizations (SMOs) characterized by a horizontal management structure as an effective alternative to existing hierarchical management structures overladen with bureaucracy. Therefore, we are exploring the strategies at self-managed homecare organizations that can make nursing a better and more productive experience.

**Method:**

This study utilized Constructivist Grounded Theory (CGT), employing semi-structured interviews to explore nursing dynamics in horizontal organizational structures. It delved into crucial aspects like finances, organizational structure, value systems, information flow, and conflict resolution within SMOs. The methodology involved theoretical sampling, prioritizing expert self-management knowledge over mere representativeness. Seven nurses, twelve management members, and fifteen patients from self-managed homecare organizations contributed to the examination of nursing experiences. Constant comparative analysis of data led to the identification of the Qualitative Success Enablers (QSEs), revealing three themes: Insightfulness, Enhancing Nursing Experience through Job Enrichment, and Autonomy-Enabled Intrapreneurship.

**Results:**

The findings indicate that the horizontal management structure represented by the studied organization in India has shown considerable success in times laden with uncertainties during the COVID-19 pandemic, especially during the delta wave, which revealed the frailty of existing healthcare infrastructure. The organization successfully maintained a better nursing experience and gained patient and employee satisfaction, as revealed by in-depth semi-structured interviews and constant comparative analysis.

**Conclusion:**

In a world of unique challenges, we stand on the brink of significant transformations. SMOs are vital in India’s homecare sector for enhancing nursing experiences and overall organizational performance. Fostering a trust-based environment within SMOs is integral to delivering effective services. The autonomy to design nursing jobs, insightfulness, and innovativeness in the nursing job through suitable training activities, various job enrichment methods, and finding meaningfulness in a job through softer aspects of caregiving result in an enhanced nursing experience at SMOs. This groundbreaking approach can be extended to other homecare organizations in India, relieving the strain on the existing healthcare system.

**Supplementary Information:**

The online version contains supplementary material available at 10.1186/s12912-023-01647-5.

## Background

### Nursing sector and challenges in India

Even though nursing has evolved into a profession, according to the World Health Organization (WHO), nurses often find themselves in a subordinate position within the medical fraternity [1]. Nurses are crucial in fostering collaboration among healthcare providers, including doctors, paramedical staff, and support personnel. However, they grapple with many daunting challenges in their workplace, which hamper their efficiency in delivering quality patient care and tarnish the reputation of the healthcare institution they serve. Notably, these challenges are frequently cited as the primary reasons motivating nurses to exit the profession, resulting in a need for more nursing students and exacerbating staff shortages in sectors like homecare. These challenges are intricately interlinked, and their repercussions are far-reaching.

#### Hierarchy as a Barrier in Nursing

The nursing profession’s hierarchical structure impedes nurses’ career advancement. Opportunities for promotion are often limited and delayed due to infrequent Departmental Promotional Committees, which hinder nurses’ professional progression [2]. The rigid hierarchical structure restricts role rotation, preventing nurses from gaining diverse experiences and effectively contributing to patient care [3]. The absence of clear career pathways and training opportunities exacerbates the issue, as nurses require support in acquiring the skills and knowledge needed to deliver high-quality care.

#### Functional barriers

The absence of well-defined job roles and responsibilities for Nurse Practitioners (NPs) leads to clarity and efficiency in healthcare delivery. Improved collaboration among healthcare team members could enhance efficiency, yet most of the time it is not achieved, adversely affecting patient care [4]. Medico-legal uncertainties contribute to professional stress among nurses, impacting decision-making and the quality of patient care [5]. Furthermore, more mentoring and supervision are needed to ensure nurses’ professional development and maintain care quality [6].

#### Perceptional barriers

Perceptional barriers play a significant role in the challenges faced by the nursing sector in India. Research indicates a need for an understanding of nursing curriculum, training, and the roles of advanced nurse practitioners among management, including doctors and other healthcare team members [7]. This lack of understanding hampers collaboration and recognition of nurses’ capabilities. Job and financial security concerns among general practitioners create resistance to nurses assuming roles traditionally performed by the managers [8]. The negative public image of the nursing profession discourages individuals from pursuing nursing careers, insinuating a need for finding meaningfulness in the nursing job, while a lack of public awareness about the education and role of nurse practitioners limits their acceptance and utilization [9, 10].

#### High turnover rates

High turnover rates among nurses in India exacerbate the aforementioned challenges. Poor leadership styles, long working hours, inadequate salaries, and inconsistent work environments contribute to a high nurse turnover due to degraded nursing experiences [11]. These turnovers result in patient dissatisfaction, compromised patient safety, and a decline in the reputation of healthcare institutions [12].

Implementing comprehensive reforms in organizational structures, altering public perceptions of the nursing profession, and enhancing the training of nursing practitioners are essential steps to empower nurses. Addressing these discussed challenges within the Indian nursing profession is imperative for enhanced nursing experiences for a sustained enhancement of healthcare services, particularly in the burgeoning homecare sector in India.

### Self-management in organizations (SMOs)

Definition: SMOs radically decentralize authority while retaining a formal organizational structure with minimal need for supervision, fostering autonomy and decision-making at all levels, and distributing responsibilities among individuals or teams. This shift from traditional hierarchy empowers employees to take ownership, collaborate, and innovate, exploring less-hierarchical structures for adaptability and engagement [13, 14].

“Self-management” in organizations involves implementing organizational principles aimed at decentralizing power, clearly outlining roles and tasks, and providing as many discretionary powers as possible to all team members [15]. Recognizing individual differences in strengths, weaknesses, interests, and resilience is fundamental to self-management [15, 16]. It is a tenet of self-management to ensure that all members of an organization have an equal voice in formulating and enforcing policies and procedures and distributing power openly and honestly [17, 18]. People in SMOs do not answer to a higher authority but instead abide by the rules and agreements they have created, and therefore, the need for a rigid superior-subordinate dynamic is eliminated. It involves running an organizational system that rejects the traditional line of command, favoring more decentralization of power [19, 20]. This necessitates eliminating unnecessary management layers and giving all employees an equal say in major organizational decisions [13, 21].

In SMOs, leadership is not tied to a specific position or function; therefore, there is no necessity for senior management roles that are assumed to be filled by leaders with extensive training, experience, and corresponding high salaries [22, 23]. When people are trusted to take charge of their own jobs, they can take the reins or follow the lead depending on the dynamic circumstances at the organization [22–24].

The following Table 1 shows the characteristics and advantages of SMOs.

**Table 1:** Characteristics and advantages of SMOs

Characteristics	Meaning	Advantages
Radical Decentralization of Authority	People in self-managing organizations need not answer to a manager with broad discretion over their workdays, including assigning tasks, overseeing their completion, and setting their salaries and advancement prospects.	By doing away with traditional management structures, self-managing companies can sidestep the power dynamic inherent in a position of authority. Even though “managers” may no longer have a formal role in self-managing organizations, management is still performed without managers. Monitoring progress toward organizational goals, establishing organizational structures, and offering feedback to employees are still crucial to the success of SMOs. In SMOs, these powers are delegated formally to individuals in a fashion that is neither permanent nor boundless nor tied to any particular position in the organizational structure.
Formal System	A self-managed organization represents a formal structure that specifies how power is distributed within the organization.	For example, Morning Star, a Woodland, California-based agribusiness and food processing company, formalized its approach by outlining organizational principles for how employees should interact with one another and a method for resolving workplace problems known as the “Gaining Agreement” procedure. Morning Star established a self-management institute as a think tank and educational institution to “define, refine, and promote the ideas and instruments of self-management in organizations.”
Pan Organization	In a self-managed organization, decentralization is not confined to the frontline staff or a specific team. The statutory regulations bind all employees, from the lowest-level workers to the highest-ranking executives.	For instance, the explicit norms defining Zappos job authority apply to entry-level employees and C-suite executives. Morning Star also has a policy where all employees, including the CEO, can and do enter bilateral contracts. At Valve, CEO Gabe Newell has no more power than any other developer to decide which games get made.

### Problems faced by the SMOs and the current scenario

Some SMOs are purported to encounter a range of challenges that influence their operational efficiency and overall effectiveness. One notable challenge arises from the absence of a clear communication structure within self-managed teams. This deficiency may result in redundant communication, hindering the team’s efficiency and potentially leading to information overload [20]. Self-managed teams often face difficulties in handling conflicts. The absence of a traditional hierarchical structure may contribute to conflict resolution and decision-making challenges. This can impact team dynamics, potentially causing disruptions and affecting overall team effectiveness [13]. Without a centralized authority to manage and coordinate tasks, the team may struggle to synchronize efforts, impacting its overall performance and ability to achieve collective goals [14]. While diversity is valuable, certain forms of diversity can pose challenges for self-managing teams. Differences in perspectives, work styles, and approaches may undermine team cohesion and performance, introducing an additional obstacle for the team to navigate [22, 23]. Also, self-management frameworks may lack clear guidance on prioritization, budgeting, and resource management. This absence of direction can contribute to organizational challenges, making it difficult for teams to allocate resources effectively and align their activities with broader strategic goals [24].

## Knowledge gap

The research on nursing experience in self-managed homecare organizations in India faces several notable gaps. Firstly, there is a significant void in the literature as no prior research has delved into the nursing experience within these organizations, hindering an understanding of how nurses navigate their roles, challenges, and dynamics within self-managed structures in the Indian context [19]. This gap limits the knowledge base concerning the unique aspects of nursing practice, job satisfaction, and overall experiences in the specific context of self-managed homecare organizations in India [10, 11].

The literature lacks exploration into the impact of horizontal organizational structures, particularly self-managed structures, on the nursing experience in homecare organizations in India [20, 21]. The absence of research in this area means there is a deficiency in knowledge regarding how these structural elements influence nurses’ autonomy and the organization’s overall effectiveness, thereby impeding a comprehensive understanding of organizational development and operational efficiency in homecare settings in India.

Additionally, the research lacks a holistic perspective from all stakeholders concerning the nursing experience in self-managed homecare organizations in the Indian context [22, 23]. The limited exploration of perspectives from nurses, patients, administrators, and other relevant stakeholders hampers a thorough understanding of the multifaceted challenges and opportunities faced by nurses in self-managed homecare settings in India [24]. Furthermore, while horizontal structures may exist, prior studies haven’t specifically investigated the formal structure of SMOs with all essential characteristics in India. This gap inhibits a nuanced understanding of the organizational framework necessary for effective self-management practices in the Indian context.

The absence of a replicable model or guide for Indian organizations, particularly in the homecare sector, attempting to transform into SMOs, hampers the successful adoption of self-management principles, hindering the potential enhancement of nursing experiences in the Indian context. Lastly, the integration of technology in self-managed homecare organizations in India remains unexplored in previous research [10, 17]. This gap signifies a lack of understanding of how technological integration influences nursing practice, patient care, and overall healthcare outcomes in the Indian context, leaving a crucial aspect unaddressed in the existing literature.

## Research questions

1. How do horizontal organizational structures influence the nursing experience in the Indian homecare sector?
2. How do nurses, patients, and other stakeholders perceive the nursing experience in self-managed homecare organizations in India?
3. What strategies can be informed by the stakeholders’ insights that can be implemented to enhance the nursing experience at the homecare SMOs?

## Research aim

Horizontal organizational structures are touted to be one of the most effective organizational structures in homecare set up in European countries like the Netherlands, where homecare is a part of public healthcare services. This research explored the implications of applying horizontal organizational structures to homecare setups in India, specifically focusing on the nursing experience. We aim to explore if the horizontal organizational structures in general and self-managed organizational structure, in particular, are an answer to management inconsistencies in homecare set up in India, especially when the Delta wave of the COVID pandemic revealed the frailty of healthcare setup in India with collapsing supplies and insufficient occupancy given the population size. The analysis of interviews sheds light on the complexities and nuances that influence organizational outcomes. This will help populate the homecare ecosystem by improving the nursing experience in India, starting from the population section that can afford these services and is in need of homecare delivery, with urbanization and migration as the current reality.

## Significance of the study

This study on the nursing experience in self-managed homecare organizations in India is multi-facetedly significant. Addressing the geographical gap in research is crucial as it provides valuable insights into the unique challenges faced by nurses in self-managed homecare organizations, therefore, the authors studied the nursing experience in SMOs in the Indian context to understand the unique strategies and their importance in Indian SMOs. This study is fundamental for understanding how a different organizational decision-making can significantly improve the working conditions, enhance job satisfaction, and ultimately influence positive patient care outcomes in self-managed homecare organizations in India.

Investigating the impact of horizontal organizational structures on the nursing experience in the Indian homecare sector holds great significance. This exploration is essential for fostering organizational development and ensuring the effectiveness of self-managed structures. Insights gained from this research inform strategies that optimize the structural elements of homecare organizations, promoting efficiency and effectiveness. Obtaining a holistic stakeholder perspective in the Indian context is significant for a comprehensive understanding of the nursing experience. Insights from nurses, patients, administrators, and other stakeholders are helpful in addressing challenges and leveraging opportunities within self-managed homecare organizations, contributing to a more robust healthcare system in India with enhanced nursing experience.

Understanding the formal structure of SMOs in India is critical for implementing effective self-management practices. This knowledge contributes to organizational efficiency, creating an environment conducive to nursing excellence and overall success. This study provides replicable strategies for Indian organizations to adopt self-management principles, particularly in the homecare sector. This study has the potential to enhance nursing experiences, contributing to the overall success of organizations seeking to transform into self-managed entities. Investigating technological integration in Indian self-managed homecare organizations is essential for leveraging the benefits of modern technology. This research can inform practices that improve the nursing experience, patient outcomes, and the overall healthcare landscape in the Indian context.

## Method

### Study design

In exploring the nuanced dynamics of nursing experiences within the Indian self-managed homecare sector, this qualitative study was meticulously designed through a constructivist grounded theory lens [25–28]. CGT is relevant when the research is needed to explain the actions and processes of a specific situation in a given context [25]. Diverging from traditional grounded theory, which seeks a singular objective truth, CGT appreciates the subjective nature of reality. Therefore, it allows the authors to accommodate more than one core categories or themes as they emerge in data analysis, and authors do not need to limit or shrink their findings to a single insufficient theme [25]. This methodology exhibits distinctive features, beginning with its inductive approach—forging multiple findings organically from data rather than relying on preconceived notions and, therefore, proposing a theory is not mandatory [25].

Crafted to offer profound insights, the research design in this study delved into the perspectives of crucial stakeholders—devoted nurses, care recipients (patients), and management members selected through theoretical sampling [29]. Beyond understanding nurses’ perspectives for exploring the nursing experience at the homecare SMOs, the interviews extended to include patients receiving homecare services, providing insights into the nursing experience. While the management members’ perspectives illuminated the functioning of SMOs in Indian homecare. This study was a longitudinal study [30] and the comprehensive interview period commenced with engaging management members and extended over 2 years, spanning from August 2021 to August 2023. This longitudinal approach allowed for a nuanced understanding of the evolving dynamics within the studied self-managed homecare organization.

## Research setting

### Buurtzorg Edugreen

The authors intended to study nursing experience at the self-managed homecare organizations in India. Therefore, the authors selected ‘Buurtzorg Edugreen’ for this study to understand and explicate the nursing experience at SMOs in the Indian context. The gatekeepers at Buurtzorg Edugreen agreed to provide the authors with organizational data access and arranged the interviews with the interviewees. Buurtzorg Edugreen is a joint venture between Buurtzorg Asia, Edugreen India, and other shareholders [31]. It is a patient-centric, self-managed home care organization, and the profit element is kept in mind just to keep the operations going.

## Participants

Seven nurses, fifteen patients, and twelve management members of the studied SMO were interviewed. Through email communications and virtual meetings, rapport was built with the organizational gatekeepers, the CEO, the Directors, and the Operations Head. Mutual trust was built with organizational members so that the required data could be exchanged freely and the interviewees could be contacted without them holding back any vital information from the interviewer. The nurses interviewed had prior experience in nursing before joining the studied SMO. The mean age of nurses was 26.8 years. Patients interviewed were between 78 and 91 years of age. Their mean age was 85.6 years. The management members interviewed had five to 25 years of experience in the healthcare and training sector. They were aged between 33 and 53 years, and their mean age was 41.3 years.

## Interview guide

A semi-structured interview protocol was developed based on existing literature on self-management, horizontal organizational structures, the status of homecare in India, and self-managed homecare organizations. Nurses were interviewed concerning their working experience (employees’ perspective) at the SMO. The patients were interviewed using a semi-structured group of questions [32, 33] regarding their opinions on the quality of service. Patient interviews proved to be valuable tools for revealing nursing experiences, as they provided insights into the quality of care and interactions between nurses and patients. The management members were interviewed to get an idea about their motivation for bringing the concept of self-managed homecare in India and how this management structure affects the nursing experience in the organization. A typical would last between 35 minutes to one and a half hours. All interviews were recorded using a voice recorder, and the voice recordings were later manually transcribed.

Some sample questions included in the interview protocol are: (a) Do you feel a greater sense of independence with this organization? If yes, how? (b) Do you find a great deal of coordination among nurses? Please tell us something about the coordination among the nurses. (c) Is being directed by the management better, or is being autonomous more conducive to your job delivery? How is it so? (d) What changes can the self-managed homecare organizations make to ease care delivery and improve the quality of care provided? (e) Do you feel trusted at your homecare organization? If yes, how does it help in enhancing job performance? If not, why?

## Data collection

The data was collected in alignment with the constructivist grounded theory approach. Measures were taken to assure the interviewed patients’ families that the interviews would not disrupt ongoing patient care, emphasizing flexibility in halting the interview if any discomfort arose. A commitment to maintaining confidentiality and preserving dignity throughout the interview process and afterward was explicitly communicated to the families. The authors ensured nurses’ anonymity and assuring that their input would not adversely affect their careers was a pivotal aspect of the ethical considerations. The patients, displaying generosity, granted the authors access to their homes, allowing for interviews that fostered trust through genuine introductions and a transparent explanation of the study’s purpose. The unique setting of interviewing nurses within patients’ homes facilitated an in-depth exploration of in situ service delivery, capturing close interactions between nurses and patients. Despite the recorded interviews lasting between 35 minutes to one and a half hours, the researcher’s extended presence (3 to 5 hours per home) accommodated interruptions for essential nursing activities and caregiving responsibilities. CGT guided the repetitive collection and analysis of data until reaching saturation, ensuring a thorough exploration till no new data emerged [34]. The data was collected over a period of 2 years.

The data collected was ‘rich’ with a carefully and comprehensively crafted interview guide discussed in a previous section with close reference to the self-managed homecare organizations in the Indian context and the global existing literature on self-managed homecare organizations. The authors used theoretical sampling, selecting participants based on emerging categories and concepts. This ensured ongoing relevance to the research question and contributed to the achievement of data saturation. At the end of data collection, the authors had thirty-four participant interview recordings. Therefore, each of these interviews yielded the data that was many-layered, intricate, and detailed. At the same time, the data was ‘thick’ with a lot of explanations and expressions of emotions by all the participants owing to the interviewing skills and trust building by the interviewee among the participants. Therefore, the data was rich and thick [32], as the interviewee focused on obtaining intricate and substantial information through extensive interviews and detailed descriptions supported by a robust and suitable interview guide [34, 35]. This approach accelerated the saturation process [34] by uncovering nuanced details and facilitating the emergence of new themes. The data could answer all the research questions sufficiently, therefore enabling replicability [34, 36] of the study in similar contexts.

## Data analysis

**Fig. 1:**
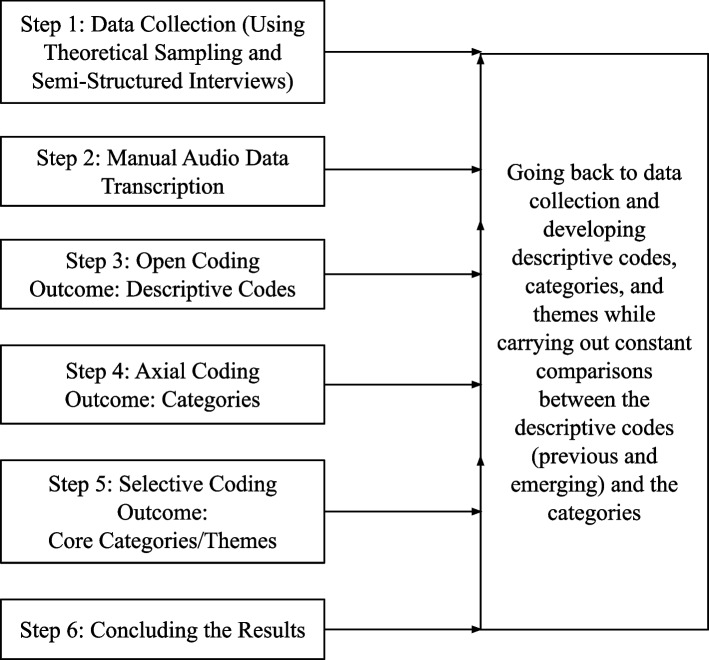
Diagrammatic representation of the constant comparative data analysis process as per CGT

Figure 1 below gives an overview of the data analysis process adopted in this study following CGT.

The audio recordings of the interviews were manually transcribed verbatim and de-identified by the first author. Constant comparison was used as the method in the data analysis process. The coding process was based on theoretical sampling, coding, constant comparison, identification, and data saturation [36, 37] in line with the CGT approach, which includes a reflexive, repetitive involvement with the dataset to produce a solid analysis till the point when no new information emerges from further data collection. The data was simultaneously collected and analyzed over 2 years till the saturation point in this concurrent data collection and analysis process.

The analysis process started by reading the transcription openly several times to become familiar with the dataset. The authors then re-read the transcription with the aim in mind and started the coding process, where they focussed on organizing data meaningfully and systematically, marking the descriptive codes that emerged through initial open coding [38, 39]. Also, the authors took up any point of disagreement and discussed. Weightage was given to the interviewer who conducted the interview.

The authors then examined the descriptive codes and performed axial coding by developing and reviewing the code data and the dataset, checking whether the codes fit together and whether they answered the research questions. The axial coding yielded broader categories by establishing relationships and connections between descriptive codes. Axial coding involved grouping similar codes together and creating broader categories [38–41]. The authors communicated constantly regarding the emerging broader categories and reached the final broader categories after mutually agreeing.

In the final data analysis stage, the first author performed selective coding to identify core categories or themes that integrated and explained the relationships between the related categories [38, 39]. The categories identified during axial coding were grouped into relevant themes. The three themes were Insightfulness, Enhancing Nursing Experience through Job Enrichment, and Autonomy-Enabled Intrapreneurship. The second author agreed on the emergent themes after going through the selective coding process performed on the data by the first author. These three themes and eight categories encompassed all the overlapping and new descriptive codes that emerged during the iterative analytical process. The themes and categories were subsequently defined, and findings were written as the authors reported in the results section of this paper.

## Techniques used by authors to enhance trustworthiness

### Integration of member checking and Audit Trail

The authors employed a combination of member checking and a well-maintained audit trail that reinforced the credibility and dependability of this study. It allowed us to track the evolution of authors’ interpretations and the extent to which they aligned with participants’ experiences. The authors’ commitment to both member checking and an exhaustive audit trail underscores the dedication to rigor and transparency in this qualitative nursing research, further solidifying the trustworthiness of our findings.

### Triangulation of data

Incorporating both member checking and an audit trail allowed the authors to triangulate the data. This multi-pronged approach enhanced the confirmability and trustworthiness of this research. Integrating member checking and audit trails as complementary strategies for data validation and documentation increased the robustness of research findings and holistically added layers of trustworthiness to this research.

## Results

The following Table 2 shows the participants’ demographics and the codes used to mention them in this paper to maintain anonymity.

**Table 2:** Participant information coding for anonymity

Patient/ Nurse/ Management Member Number	Participant Category	Gender	Age (In years)	Participant Code
1	Nurse	Female	Thirty-Four	N-1-F-34
2	Nurse	Female	Thirty-Three	N-2-F-33
3	Nurse	Female	Twenty-Four	N-3-F-24
4	Nurse	Female	Twenty-Six	N-4-F-26
5	Nurse	Female	Twenty-Three	N-5-F-23
6	Nurse	Female	Twenty-One	N-6-F-21
7	Nurse	Female	Twenty-Seven	N-7-F-27
1	Patient	Female	Eighty	C-1-F-80
14	Patient	Female	Eighty-Nine	C-14-F-89
2	Management Member	Male	Fifty- Three	MM-2-M-53
4	Management Member	Male	Fifty-One	MM-4-M-51
5	Management Member	Male	Fifty	MM-5-M-50
8	Management Member	Male	Thirty-Six	MM-8-M-36

The data revealed the “qualitative success enablers” as the emergent themes and categories. We define the term “qualitative success enablers” as follows:

“Qualitative success enablers” in a self-managed homecare organization are factors, strategies, or elements that enhance the quality of nursing care and the overall nursing experience. They emphasize subjective and qualitative aspects of success rather than quantitative measurements and profoundly impact the nursing profession and care delivery in a home setting.

The final themes and categories have been briefly described in Table 3. The subsequent sections describe the themes in detail with prototypical excerpts.

**Table 3:** The emerging themes, categories, and indicators from the constant comparative analysis method

S. No.	Major Themes	Categories	Description
1	Insightfulness - *Insightfulness in the context of nursing experience refers to the capacity of nurses to apply advanced critical thinking abilities, spontaneous insightful and innovative solutions, and creativity in their day-to-day jobs, offering immediate as well as long-term approaches to patient care. It encompasses integrating diverse perspectives, including fairness, ethics, and evidence-based practice, into their decision-making and problem-solving processes. The nurses at SMOs are enabled to apply insights in day-to-day jobs spontaneously as and when the need arises, given they are trusted and have enough freedom to be innovative.*	1. The Freedom to Experience Meaning	This concept challenges the conventional belief that work’s meaning is solely determined by individual viewpoints, highlighting the profound importance of a shared sense of meaningfulness among colleagues. It underscores that in SMOs, nursing is not confined to purely career-driven motivations; rather, it acknowledges satisfaction from tending to the compassionate and softer aspects of caregiving for a better nursing experience along with being insightful through the freedom to apply critical thinking skills in their day to day jobs as nurses. This counters the bias towards a purely career-centric approach, emphasizing the value of more profound meaning and personal satisfaction in the workplace derived from being insightful and creative. It demonstrates the nurses’ problem-solving capacities as they do not feel excessively controlled and micro-managed to perform their tasks rigidly.
		2. Expansive Nursing Intelligence	Expansive Nursing Intelligence embodies the nurses’ capacity to employ advanced critical thinking skills and insights that transcend the confines of clinical duties, embracing a comprehensive perspective on homecare. Its goal is to improve the holistic care provided to homecare patients, thereby improving their overall well-being and outcomes. This, in turn, elevates the nursing experience, fostering recognition and career advancement for nurses.
2	Enhancing Nursing Experience through Job Enrichment - *SMOs involve this unique approach to achieving organizational excellence. These strategies encompass integrating additional job support, cultivating a culture marked by group harmony and cooperation, utilizing compact and specialized teams, role rotation, and a dedicated effort to nurture critical thinking among employees as a means of job enrichment. These ways of job enrichment contribute to enhancing the nursing experience at SMOs.*	1. Assisting in supplementary jobs	Assisting in supplementary jobs through the utilization of Information and Communication Technology (ICT) solutions, like the Dr. Dashboard software developed by GL Tech, leads to job enrichment and enhances the nursing experience at the SMOs. These ICT tools alleviate the administrative burden by enabling nurses to efficiently manage tasks beyond their core caregiving responsibilities, such as recording patient information, health data, treatment records, nursing information, and caregiver details. Moreover, they facilitate seamless communication and data sharing among the team. This, in turn, enhances nurses’ job enrichment, satisfaction, and nursing experience and contributes to the organization’s growth and efficiency.
		2. Rotating Team Duties	“Rotating Team Duties” is a progressive approach adopted by Self-Managed Organizations (SMOs) to ensure job enrichment in self-managed homecare organizations. In this practice, team members are empowered to express their interest in and assume particular responsibilities within the team, breaking away from the conventional belief that duties should be rigidly predefined and assigned based on predetermined roles. In SMOs, team members have the autonomy to adapt and distribute duties according to their needs and preferences, all while ensuring that essential tasks are effectively accomplished. This flexible approach fosters a dynamic and adaptive task allocation system within the organization, which, in turn, contributes to job enrichment and a better nursing experience.
		3. Small Team Size	Implementing a “Small Team Size” approach in a self-managed homecare organization entails deliberately maintaining a limited number of team members, often capped at a maximum of two individuals. This strategic choice is instrumental in enhancing nurses’ job performance. It enriches their job by paying attention to softer aspects of caregiving as they provide homecare to the same patients over long periods, leading to a superior nursing experience. It fosters a patient-focused approach, ensuring a more personalized and attentive care experience. This patient-focused approach also enriches the job experience of nurses as they are able to provide more humane care, focusing on softer aspects like empathy, compassion, and genuine concern for patients’ well-being while performing their caregiving jobs.
3	Autonomy-enabled Intrapreneurship -*"Autonomy-Enabled Intrapreneurship” in the context of SMOs signifies a proactive and entrepreneurial mindset that empowers employees to foster innovation, take initiative, and champion transformative change within the organization through enhanced autonomy given to the nurses. This approach encourages nurses to drive progress and growth through greater discretion on designing and performing their jobs while ensuring the implementation of necessary safeguards to maintain a proactive and balanced organizational ecosystem. In nursing, autonomy-enhanced entrepreneurship promotes a superior nursing experience by allowing healthcare professionals greater independence, autonomy, and control over their work. It promotes nurse leadership, ultimately leading to improved patient care, job satisfaction, and overall organizational excellence.*	*1. Protecting and Expanding Organizational Ecosystem*	“Protecting and expanding organizational ecosystem” entails preserving and enriching a distinctive work culture through training and deconditioning, promoting a collaborative and creative atmosphere for open expression, and retaining employees who align with the organization’s values. Accepting natural departures for those who don’t fit the ecosystem avoids immediate firing, reducing job insecurity and fostering a respectful and supportive work environment, ultimately enhancing the nursing experience.
		*2. Safeguarding and Fostering Freedom Parallel to Safeguards*	“Safeguarding and Fostering Freedom in parallel with Safeguards” signifies a guiding principle within SMOs that underscores the significance of striking a delicate equilibrium. It involves providing employees, especially those in roles that demand critical thinking and expertise, with the freedom to make autonomous decisions while concurrently establishing protective measures to avert unwanted outcomes. This approach acknowledges that curtailing this freedom may result in adverse consequences, including ethical dilemmas, coordination challenges, diminished motivation, reduced care quality, and overall dissatisfaction among nurses, therefore leading to a better nursing experience. Consequently, it recognizes the indispensability of both granting autonomy and implementing safeguards as fundamental tenets of the organization’s core values, ultimately leading to a more rewarding nursing experience.
		3. Leadership in Nursing	“Leadership in nursing” at SMOs encompasses the proactive and unwavering commitment demonstrated by nurses within SMOs, to advocate for social justice, fairness, and positive change. It entails taking a leading role in efforts aimed at achieving justice and inspiring transformative actions that ultimately enhance the nursing experience. Nurse leaders are motivated by their core principles and values, driven by a profound sense of duty, a strong belief in the significance of taking action, and an enduring commitment to improving the healthcare environment. Essentially, it signifies a determined dedication to causing positive change and making a meaningful impact that extends beyond their immediate caregiving responsibilities, ultimately resulting in a better nursing experience.

## Insightfulness

A key construct to the context of nursing experience at self-managed homecare organizations was *Insightfulness.* It demonstrates the capability of nurses to employ advanced critical thinking, spontaneous innovation, and creativity in their daily tasks, presenting both immediate and long-term solutions to patient care. This involves integrating diverse perspectives, such as fairness, ethics, and evidence-based practices into decision-making and problem-solving processes. Insightfulness allows nurses at self-managed homecare organizations to discover profound meaning in their work, transcending mere career aspirations. It encourages a compassionate and fulfilling caregiving approach, where nurses derive meaning in their day-to-day roles. The broad intelligence within nursing promotes insightful decision-making in addressing challenges specific to homecare. This holistic perspective enhances patient well-being and elevates the nursing experience, opening doors for recognition and career advancement.

### Freedom to experience meaning and purpose in work and at the workplace

A fundamental principle within SMOs is empowering employees to discover personal and collective significance in their work that transcends individual perspectives. This concept challenges the conventional belief that work’s meaning is solely determined by individual viewpoints, highlighting the profound importance of a shared sense of meaningfulness among colleagues. It underscores that in SMOs, nursing is not confined to purely career-driven motivations; rather, it acknowledges that satisfaction and fulfillment can be derived from tending to the compassionate and softer aspects of caregiving for a better nursing experience along with being insightful through the freedom to apply critical thinking skills in their day to day jobs as nurses. Nurses become more insightful and creative through the demonstration of their problem-solving capacities as they do not feel excessively controlled and micro-managed to perform their tasks in rigid ways.

Employees who serve as role models in SMOs share their knowledge and radiate “love and joy in what they are doing,” acting as catalysts for meaningful experiences. At the studied SMO, nurses firmly believe that “people’s achievements are so celebrated here.” This Qualitative Success Enabler challenges the assumption that meaningfulness at work is solely shaped by individual perception, focusing on the shared perspective of nurses at the studied SMO. The collective perception of nurses significantly contributes to their experience of meaningfulness at work. The nurses’ satisfaction is derived from applying insightful ways to problem solving as and when encountered as nurses are deconditioned to perform jobs and solve problems spontaneously with their agile approach. It implies that in SMOs, the profession extends beyond career choices, encompassing deeper meanings and personal fulfillment.

The nurses’ perception of experiencing meaningfulness at the SMOs is expressed in the following statement,

*The work you put in is valued. I think that all any of us ask for is to have our efforts valued. I had N number of options for a career, yet I chose the nursing profession as it brings out my compassionate side that values human service. I feel happy when a woman aged as my grandmother holds my hand every day; she wakes up in the morning and kisses my cheeks with her hands on my head to give her blessings. Nothing can compare to the joy of this feeling and the satisfaction it brings to me. At this homecare organization, I am free to apply my insights and creativity into the day-to-day jobs, and that’s where I derive satisfaction and meaning in life from the nursing profession. (N-5-F-23).*

*Descriptive code: Gratitude in Action- Embracing Compassion in Nursing.*

Nurses at SMOs are not only heard but actively engaged in reflection and shared decision-making. They receive support and access to learning opportunities. Autonomy and engagement contribute to meaning and joy in nursing practice, further enhancing the nursing experience. One nurse shares her journey,

*I feel happy to care for the ailing and help them heal. Apart from the freedom to perform jobs insightfully through the application of my creativity and freedom to design my job spontaneously as suited to the context, I have learning and higher education opportunities here. This homecare organization supports further educational qualifications like GNM, B.Sc. Nursing and whichever qualification we may like to pursue for our career growth and skill development through flexible working hours and no pay cuts in case of leaves for such educational pursuits. Yes, I feel fulfilled in this homecare organization and have a sense of purpose in my life. (N-7-F-27).*

*Descriptive code: Fulfillment in Nursing- A Holistic Approach to Career Growth.*

Another nurse adds,

*In this organization, nurses don’t just share knowledge but also exude love and joy in what we do. This creates a culture where people’s achievements are celebrated. This approach challenges the idea that meaningfulness at work is solely shaped by individual perception and emphasizes the shared experience of meaningfulness in work. In this organization, I am able to value my compassionate side and find satisfaction in insightful problem-solving instead of being solely career-driven. Through this job, I am able to fulfill my career aspirations to embrace deeper meanings and personal fulfillment in the nursing profession. (N-3-F-24).*

*Descriptive code: Cultivating a Culture of Shared Meaningfulness in Nursing.*

This Freedom to Experience Meaning adds depth to the nursing profession and fosters a shared sense of purpose and fulfillment among nurses, resulting in a more rewarding nursing experience.

#### Expansive nursing intelligence

Expansive Nursing Intelligence embodies the nurses’ capacity to employ advanced critical thinking skills and insights that transcend the confines of clinical duties, embracing a comprehensive perspective on homecare. Its goal is to improve the holistic care provided to homecare patients, thereby improving their overall well-being and outcomes. This, in turn, elevates the nursing experience, fostering recognition and career advancement for nurses.

Traditionally, the presumption was that healthcare professionals, including nurses, were dedicated solely to their assigned tasks. However, the performance indicators at SMOs challenge this notion, revealing that nurses strive for excellence by exceeding their allocated responsibilities. They perform multiple tasks during a single shift - from changing wound dressings to providing prescriptions and monitoring vital signs. This requires critical thinking skills and the application of insights for solving day-to-day patient problems effectively. It beckons nurses to explore a multitude of viewpoints encompassing fairness, ethics, and evidence-based practice. SMOs exemplify this philosophy, demonstrating how nurses think beyond their designated roles to deliver unparalleled patient service.

Nurses operate in a dynamic environment, often juggling the care of multiple patients. Critical thinking is their compass, ensuring patient safety and well-being while orchestrating a symphony of tasks. Even those who do not possess innate critical thinking skills are meticulously trained to acquire this invaluable competence. At SMOs, critical thinking comes to the fore as nurses navigate the ever-shifting landscape of patient needs. This is clear from the following statement by a nurse:

*I use critical thinking to select which medications to provide and how to organize my day caring for patients. The situations and settings of patients are in constant motion. To keep their patients safe and satisfied, I constantly analyze and reevaluate the information I acquire. (N-4-F-26).*

*Descriptive code: Utilizing Critical Thinking in Patient Care.*

The COVID-19 pandemic highlighted the necessity of critical thinking in home care scenarios, particularly for intensive care nurses. It was a time when nurses were called upon to employ their critical thinking skills to their fullest extent, ensuring quality care while conserving precious resources. The following statement by a nurse testifies the same:

*The COVID-19 pandemic necessitated critical thinking in homecare scenarios. It was required of intensive care nurses. Throughout the pandemic, nurses were urged to engage in critical thinking. As a nurse in intensive care, it was a test of my previously held views and my ability to provide quality care while conserving resources. (N-3-F-24).*

*Descriptive code: Necessity of Critical Thinking in Homecare and Intensive Care during COVID-19.*

One nurse aptly captures the essence of nursing intelligence, stating,

*I, as a nurse providing homecare, am frequently the first to identify problems because I am by the patient’s bedside. I collect the necessary subjective and objective patient data by formulating a brief problem statement or inquiry for the physician or advanced practice provider. (N-6-F-21).*

*Descriptive code: Identification of Subtlety of Patient Problems in Homecare.*

This Qualitative Success Enabler underscores the importance of holistic critical thinking, one that incorporates considerations of fairness, ethics, and evidence-based practice. Expansive Nursing Intelligence as a Qualitative Success Enabler improves the nursing experience at the SMOs through enhanced recognition and appreciation of nurses for their performance, gaining patient loyalty and professional development, and increasing nurses’ confidence and competence in handling diverse situations.

## Enhancing nursing experience through job enrichment

SMOs involve this unique approach to achieving organizational excellence. These strategies encompass integrating additional job support, cultivating a culture marked by group harmony and cooperation, utilizing compact and specialized teams, role rotation, and a dedicated effort to nurture critical thinking among employees as a means of job enrichment.

### Assisting in supplementary jobs

Assisting in supplementary jobs through utilizing Information and Communication Technology (ICT) solutions leads to job enrichment and enhancing the nursing experience at SMOs. These ICT tools alleviate the administrative burden by enabling nurses to efficiently manage tasks beyond their core caregiving responsibilities, such as recording patient information, health data, treatment records, nursing information, and caregiver details. Moreover, they facilitate seamless communication and data sharing among the team. This, in turn, enhances nurses’ job enrichment, satisfaction, and nursing experience and contributes to the organization’s growth and efficiency. The implementation of ICT aligns with the distinctive features of SMOs, such as decentralization and the reduction of management layers.

The following statement by a nurse unveils the impact of the use of ICT perceived by nurses:

*ICT solutions help us efficiently manage supplementary tasks like recording patient information, health data, and treatment records and save us time that we can invest in other patient-related chores. They also enable us to communicate and share data seamlessly within the team. By reducing the administrative burden and improving communication, ICT tools enrich our jobs and contribute to our better experience at this organization. (N-5- F-23).*

*Descriptive code: Leveraging ICT in Homecare Management.*

In line with the evolving trends in online training and eLearning, the studied homecare organization recognizes the importance of providing its nurses with accessible and efficient learning opportunities. This is achieved through a Learning Management System (LMS) provided by a startup based in India. This LMS allows nurses to engage in microlearning tailored to their busy schedules. Microlearning not only facilitates the absorption of significant volumes of information but also makes the learning process enjoyable, all while respecting the time constraints and commitments of the nurses in their primary role of delivering effective care to patients.

The following nurse’s statement elucidates the importance of LMS and microlearning,

*The LMS is a game-changer for our training and learning experiences. It allows us to learn even in our busy schedules. We can access short presentations and 1–2 minute videos on our smartphones, making it possible to learn on the go during commutes or personal time. This not only helps us absorb a significant amount of information but also we enjoy the learning. This approach respects our time constraints and commitments while ensuring we continue providing effective patient care. Serving our patients better gives us pride and joy in our profession. (N-7-F-27).*

*Descriptive code: Transformative Impact of ICT Tools on Learning and Patient Care.*

A management member further explains the importance of ICT through assistance in supplementary jobs,

*The nurses are busy all day commuting to the homes of patients, care delivery, and house chores. They are left with hardly any time for learning, so we tried simplifying the learning for them and, at the same time, making it enjoyable for them. We acquired the Learning Management System from Playablo Technologies based in Bangalore, India. It enables nurses to grasp the microcapsules of knowledge using brief presentations and 1–2 minutes of videos. The nurses can access such information on metro trains while walking and whenever they may find some me-time. Such LMS has made learning fun and easy for nurses. (MM-2-M-53).*

*Descriptive code: Transforming Nurses’ Learning with ICT Tools.*

The integration of ICT solutions, combined with the LMS, simplifies and enriches the learning experience for the nurses. This approach acknowledges the demanding nature of their work, offering them flexibility and convenience in their continuous professional development, ultimately contributing to job enrichment and ensuring the highest standard of care for patients through enhanced nursing experience.

### Rotating team duties

Here, the team members are empowered to express their interest in and assume particular responsibilities, breaking away from the conventional belief that duties should be rigidly predefined and assigned based on predetermined roles. This flexible approach fosters a dynamic and adaptive task allocation system within the organization, which, in turn, contributes to job enrichment and a better nursing experience.

Another statement makes it clear how rotating team duties enhance their nursing experience at SMOs,

*Rotating Team Duties enables team members to have the autonomy to express their interest in specific responsibilities rather than being tied to predefined roles. This flexibility allows us to adapt and distribute duties based on our needs and preferences while ensuring essential tasks are accomplished. This dynamic and adaptable approach within SMOs contributes to job enrichment and makes nursing more fulfilling. (N-3-F-24).*

*Descriptive code: Embracing Autonomy Through Rotating Team Duties.*

The flexibility given to nurses to express their interest in taking on specific responsibilities empowers team members, allowing them to tailor their roles to match their needs and preferences, creating a dynamic and adaptable approach. This encourages teams to reconfigure their leadership structure, demonstrating that leadership roles are not fixed and can be rotated, which reduces biases associated with hierarchical structures.

The following statement from a nurse helps us better understand the importance of the rotation of team duties,

*In this organization, nurses are free to express their interest in particular responsibilities, which goes against the traditional notion that duties are rigidly fixed. This flexibility empowers team members to tailor their roles to suit their needs and preferences, which creates a dynamic and adaptable approach. Additionally, this organization actively promotes the rotation of duties among team members, which includes leadership roles. This practice demonstrates that leadership roles are not fixed and can be rotated, helping reduce biases associated with hierarchical structures and promoting a more collaborative and flexible work environment. (N-6-F-21).*

*Descriptive code: Fostering Flexibility and Collaboration in Nursing Roles.*

The benefit of this approach is two-fold. First, it allows nurses to care for patients with varying medical cases, fostering exposure to new challenges, skills, and knowledge sets, leading to job enrichment. This contributes to professional growth and greatly enhances job satisfaction and pride. Secondly, the flexibility and freedom to express interests and tailor their roles reduce the biases stemming from rigid role assignments and hierarchical structures, leading to a better nursing experience. The following statements from nurses testify to the aforementioned benefits,

*At this homecare organization, I’ve been given numerous chances to develop my skills and learn new ones. I’ve also expanded my horizons by taking on multiple team positions and taking on responsibilities previously outside my job’s scope (such as scheduling and roster preparation). Everyone can get better at making plans here, and you may keep your skills sharp by taking on various challenging roles. (N-5-F-23).*

*Descriptive code: Skill Development and Growth at the Homecare Organization.*

*I am a GNM and have five years of experience in nursing. I am delighted that I am not stuck in managerial jobs, but my specialty is nursing and caregiving. This profession helps me derive satisfaction as I come from a very religious family. Yet, at times I feel a need to derive greater satisfaction with my job. Therefore I try to give innovative ideas to the management and my team alike. I devise new strategies and even change my duty to meet new homecare cases and expand my skill set and expertise. (N-7-F-27).*

*Descriptive code: Personal Satisfaction and Innovation in Nursing.*

Consequently, team role rotation is widely regarded as a job enrichment tool that creates a better nursing experience, creating a more engaged, motivated, and satisfied nursing workforce.

### Small team size

Implementing a “Small Team Size” approach in a self-managed homecare organization entails deliberately maintaining a limited number of team members, often capped at a maximum of two individuals. This strategic choice is instrumental in enhancing nurses’ job performance. It enriches their job by being able to pay attention to softer aspects of caregiving as they provide homecare to the same patients over long periods, leading to a superior nursing experience. The advantages are threefold: Firstly, it fosters a patient-focused approach that ensures a more personalized and attentive care experience. This patient-focused approach also enriches the job experience of nurses as they are able to provide more humane care, focusing on softer aspects like empathy, compassion, and genuine concern for patients’ well-being while performing their caregiving jobs. Secondly, it minimizes the demand for patients to interact with multiple team members, reducing complexities in communication and facilitating smoother interactions. Lastly, This approach also helps in better working terms and familiarity with the long-term team members in a small-sized team, leading to job enrichment. As a result, nurses’ job satisfaction is significantly elevated, ultimately leading to job enrichment and creating a more fulfilling nursing experience for the staff.

Maintaining a small number of nurses and nurse assistants responsible for patient care in practice promotes efficient team communication. It fosters a sense of familiarity between the nurses and the patient. This approach significantly contributes to a more rewarding nursing experience. It leads to job enrichment as nurses are able to touch the essence of caregiving through a focus on softer aspects of caregiving, as evident in the following patient statement.

*I have been cared for by the same two nurses since I joined the homecare services at this homecare organization from the very beginning. The same team of two nurses has been serving since then. I am quite satisfied as the nurses have become more like a family to me and even treat me like their mother. If it had been more nurses serving me, the same degree of warmth would not have been possible. What else could I ask for? (C-14-F-89).*

*Descriptive code: Continuity of Care and Familial Bonds in Homecare.*

Another nurse expresses how nursing experience is enhanced working with a small-sized long-term team,

*We are quite comfortable working together. We had been working as a team for the past three years and never had any problem in delivering the duty. Being familiar with each other, we know each other’s complementary skills, working styles, and behavioral needs. We have a great degree of understanding of how we can work as an effective team. We are more like sisters working together and caring for each other’s and patient’s needs. Taking care of our patients is our prime duty, and every other situation and personal comfort comes later. We both carry this understanding. (N-7-F-27).*

*Descriptive code: A Cohesive Team with Familial Bonds.*

Similar sentiments are resonated by another participating nurse on how a small team size fosters a patient-focused approach that ensures a more personalized and attentive care experience,

*Having a small team size is instrumental in fostering a patient-focused approach. The limited number of team members allows us to provide a more personalized and attentive care experience. With fewer team members involved in a patient’s care, we can dedicate more time and attention to each individual, addressing their unique needs and preferences. This approach enhances the quality of care we deliver. It strengthens the bond between our team and the patient, creating a more meaningful and fulfilling healthcare experience for the patient and our team. (N-2-F-33).*

*Descriptive code: Small Team, Big Impact on Enhanced Nursing Experience.*

This Qualitative Success Enabler challenges the conventional belief that larger team sizes are more effective in improving job performance in SMOs by highlighting the significant advantages of small team sizes. Doing so effectively mitigates the bias that assumes patients require assistance from nurses with diverse skill sets. Diverse skill sets are good to have, yet patients need nurses with skill sets that may help them in recuperating from their current health condition, and they need nurses who understand their health condition better rather than frequently assigning new nurses who are inexperienced with the patient case.

## Autonomy-enabled intrapreneurship

“Autonomy-Enabled Intrapreneurship” in the context of SMOs signifies a proactive and entrepreneurial mindset that empowers employees to foster innovation, take the initiative, and champion transformative change within the organization through enhanced autonomy given to the nurses. Autonomy-enhanced entrepreneurship promotes a superior nursing experience by allowing healthcare professionals greater independence, autonomy, and control over their work. It promotes nurse leadership, ultimately leading to improved patient care, job satisfaction, and overall organizational excellence.

### Protecting and expanding the organizational ecosystem

“Protecting and expanding the organizational ecosystem” entails preserving and enriching a distinctive work culture through training and deconditioning, promoting a collaborative and creative atmosphere for open expression, and focusing on retaining employees who align with the organization’s values. Accepting natural departures for those who don’t fit the ecosystem avoids immediate firing, reducing job insecurity and fostering a respectful and supportive work environment, ultimately enhancing the nursing experience.

The following statement by a nurse clarifies the protection and expansion of the organizational ecosystem at SMOs,

*The approach in this organization is all about preserving and enriching our unique work culture. It involves training and deconditioning to promote a collaborative and creative atmosphere. One interesting aspect is handling employees who need to align with the organization’s values. Natural departures are accepted at this organization, and we need not worry about employment. This approach enhances our confidence to be better caregivers, and the nursing job becomes more interesting. (N-4-F-26).*

*Descriptive code: Nurturing a Unique Work Culture for Enhanced Caregiving.*

SMOs prioritize the development of their nursing staff through comprehensive Orientation Training Programs. These programs empower nurses to critically assess their roles in the organization, comparing them to their previous experiences in other healthcare institutions. As part of the studied organization’s approach, nurses are encouraged to bring their unique ideas, creativity, and work ethic to deliver the most appropriate care to their patients within the specific context.

The following statement by a nurse explains how the orientation and training programs at SMOs help protect and expand the organizational ecosystem.

*These programs encourage nurses to critically assess their roles within the organization, comparing them to their previous experiences in other healthcare settings. We are able to bring our unique ideas, creativity, and work ethic into the caregiving. Here, we are not limited by conforming to established practices; instead, we are actively encouraged to contribute our ideas and innovation. This is a significant shift from traditional workplace norms, resulting in a more enriching and empowering nursing experience. (N-2-F-33).*

*Descriptive code: Nursing Empowerment through Innovative Programs.*

The primary objective of this approach is to immerse nurses in the organization’s distinctive organizational culture, ensuring that they do not carry over any issues from their previous workplaces. This approach challenges the conventional belief that employees must conform to established practices without room for their own input, creativity, and work ethics.

This Qualitative Success Enabler aligns with the overarching objective of evaluating the effectiveness of training programs and the organizational ecosystem in enhancing employee satisfaction and ensuring job enrichment in SMOs. This approach ultimately fosters a better nursing experience, where nurses are empowered to thrive and contribute their unique skills, ideas, and creativity in the service of their patients.

A management member mentions,

*If an employee does not fit in the organization, he/she can understand that himself. We ask the potential recruits to spend some time with the organization’s team at the head office in Kolkata and try to observe the way of working and culture for a week. During this period, the potential recruits are free to work and talk to anyone in the office they would like to or feel right. Usually, after a week of observation, the potential recruits can usually discern if they fit in the organization; otherwise, the universe may automatically let them know that they do not fit in and silently leave the organization. (MM-2-M-53).*

*Descriptive code: Employee Fit Assessment through Immersive Experience.*

The above statement from a management member elucidates how protecting and expanding the organizational ecosystem is achieved to enhance the nursing experience at SMOs,

*A few potential recruits even told us before deciding not to join our organization that the organization has an excellent culture and structure. However, they would not join because they think they will not be suitable for the organization as they do not have a similar mindset and attitude. We respect such clarity at our organization. (MM-5-M-50).*

*Descriptive code: Transparent Alignment Evaluation.*

### Safeguarding and fostering freedom parallel to safeguards

“Safeguarding and Fostering Freedom parallel to Safeguards” signifies a guiding principle that underscores the significance of striking a delicate equilibrium between the indispensability of granting autonomy to nurses and implementing safeguards as fundamental tenets of the organization’s core values, ultimately leading to a more rewarding nursing experience at SMOs. It involves providing employees, especially those in roles that demand critical thinking and expertise, with the freedom to make autonomous decisions while concurrently establishing protective measures to avert unwanted outcomes.

Nurses are distinguished by their intelligence, courage, and passion for their profession. They are underpinned by their critical thinking skills, ethical work practices, extensive experience, and comprehensive knowledge base, enabling them to deliver nursing care with heightened autonomy. Consequently, autonomy is highly valued by nurses at the SMOs. Autonomy, in this context, refers to the capacity of employees to engage in critical thinking and make decisions related to patient care. Restricting this autonomy can result in a range of negative consequences, including moral dilemmas among nurses, diminished nurse-physician coordination, reduced motivation in nursing roles, a sense of detachment from the caregiving process, a decline in care quality, and personal and professional dissatisfaction. Therefore, the value placed on autonomy within nursing operations is integral to the studied organization’s core principles.

A statement by nurses elucidates the approach of fostering freedom parallel to safeguards,

*In this organization, the approach is all about balancing granting autonomy to nurses and having safeguards in place. It acknowledges that we, as nurses, need the freedom to make critical decisions in patient care. However, it also recognizes that this freedom can lead to ethical dilemmas and coordination challenges without safeguards, ultimately affecting our quality of care. So, it is about valuing autonomy and safeguards equally to ensure a more rewarding nursing experience. (N-5-F-23).*

*Descriptive code: Nursing Autonomy and Safeguards Balance.*

Another nurse elucidates how this approach ensures better patient care alongside greater autonomy for the nurses to enhance the nursing experience at SMOs,

*Autonomy is essential for us nurses because it allows us to use our expertise and critical thinking skills. The organization does not see autonomy and safeguards as mutually exclusive. Instead, they emphasize the importance of balancing both aspects. We have the necessary skills and knowledge to exercise autonomy in our practice, but safeguards are in place to prevent any adverse outcomes. It is about challenging the belief that strict control is needed for effective patient care and championing the importance of autonomy and critical thinking while maintaining safeguards. This approach ensures we can make decisions confidently while providing safe, high-quality care. (N-7-F-27).*

*Descriptive code: Achieving Harmony Between Independent and Cautious Nursing.*

This approach challenges the assumption that decision-making freedom and safeguards are mutually exclusive or that one should take precedence over the other. Instead, it underscores the importance of balancing and giving equal weight to both aspects. By highlighting the need for safeguards in tandem with fostering freedom, this principle controls the bias that safeguards can be sacrificed in favor of decision-making freedom. It champions the importance of autonomy and critical thinking. These ‘safeguards’ are instrumental in preventing adverse impacts on the organization’s bottom line. They are actualized through regular patient feedback collected via telephone conversations with the head office staff, prompt resolution of nurses’ concerns by the head office to enhance their nursing efficiency, and meticulous analysis of the costs associated with caring for each patient, ensuring that profitability is maintained without compromising quality. At SMOs, the primary focus is on patients’ well-being, with profitability being a secondary consideration. In some instances, the organization may forego profitability in pursuit of the greater good for their patients. A statement from a management member at the studied SMO will further illuminate this approach.

*For our homecare organization, the patients are always the priority, and profitability comes later. There have been instances where the patients could not pay the amount required for a specific set of homecare services. In such cases, we forego profitability as we believe in retaining and maintaining compassionate patient relations. The same patients help us gain a greater patient base through word of mouth, as they are highly satisfied and happy with our services. Finally, the profitability is intact, but caring for the humane cause helps boost our organization in miraculous and varying ways. In most cases, we explain to the patient why we are charging X amount of money for Y number of days, how much the services cost, and how much is to be paid to nurses. This creates transparency in our ecosystem and attracts patients and nurses to our organization. So, we can implement flexible bureaucratic ways to create profitability. (MM-5-M-50).*

*Descriptive code: Prioritizing the Higher-Self Over Profit in Homecare Services.*

There are no further safeguards, such as monthly or quarterly reports to the CEO. This affords teams much freedom, comparable to an entrepreneur’s, leading to a fulfilling nursing experience.

### Leadership in nursing

“Leadership in nursing” at SMOs encompasses the proactive and unwavering commitment demonstrated by nurses within SMOs to advocate for social justice, fairness, and positive change. It entails leading in efforts to achieve justice and inspiring transformative actions that ultimately enhance the nursing experience. Essentially, it signifies a determined dedication to causing positive change and making a meaningful impact that extends beyond their immediate caregiving responsibilities, ultimately resulting in a better nursing experience.

This leadership encourages nurses to proactively address injustices and other triggers for taking action. SMOs are conducive to such leadership, providing an environment where nurses feel empowered to initiate change. This empowerment is further facilitated by offering training, education, and skill development opportunities. It empowers nurses to recognize their ability, choice, and authority to make a difference within the organization and society.

Leadership in nursing contributes to a better nursing experience by inspiring nurses to advocate for justice, engage in positive change, and extend their commitment to improving the health and well-being of individuals and communities. This approach fosters personal and professional growth and creates a more fulfilling and impactful nursing experience for those involved. The following statement by a management member sheds light on the same,

*Caring inevitably leads to nurses’ leadership at SMOs. When nurses awaken due to witnessing injustices and malpractices, as in the case of some homecare organizations, they can be inspired to action, and our organization is conducive to nurses’ leadership. The energy of nurses as leaders must be expended to bring about the desired transformation. (MM-8-M-36).*

*Descriptive code: Igniting Nurses’ Leadership through Compassion.*

A management member highlights the importance of nurturing nurses’ leadership through informal meetings with the following statement.

*We focus on the verbalization of the story of nurses in the informal meeting sessions with nurses. It serves as an emancipatory process that calls attention to the social, economic, and political forces affecting health, empowering them to assume a bigger role in bringing about change not only in the organization but in society at large. (MM-4-M-51).*

*Descriptive code: Elevating Nurses’ Voices for Societal Impact.*

Before leadership in nursing can evolve, individual nurses must feel empowered to take the required actions. The SMOs provide the same empowerment to the nurses, as evidenced by the following statement from a nurse.

*We are given such an environment at this homecare organization that we could recognize that we had the ability, choice, and authority to speak to those who are in senior positions in the organization. Education is a strategy for empowerment but not the sole element needed. We are provided and encouraged to join various training and education opportunities. (N-3-F-24).*

*Descriptive code: Fostering Empowerment Through Open Communication and Continuous Learning.*

A nurse practitioner also mentions how she proactively identifies and joins the training activities needed for skill development and how her organization facilitates those activities. This is clear from the following statement,

*We are provided with all the requirements for facilitating training and courses if we want to continue with further education. Education is only the beginning of learning and awareness. We, as leaders at this organization, consistently identify the importance of the educational experience in nurturing leadership that includes health policy and social justice education. (N-1-F-34).*

*Descriptive code: Fostering Leadership through Comprehensive Education.*

## Patient trust and support

Patients’ trust and confidence in the professional skills of nurses and nursing assistants emerged as an important factor for the success of the SMOs through better patient outcomes and enhanced nursing experience during data analysis. Therefore, it is essential to understand how patient trust and support are gained through the training, education, and experience of nurses at SMOs, which are attributed to acquiring the essential competencies, including innovativeness and insightfulness. This enhances the nursing experience by mutually building beneficial patient-nurse relationships. The ability of nurses to spontaneously apply advanced critical thinking skills, incorporating fairness, ethics, and evidence-based practice into their day-to-day decision-making fosters patient trust and support as it is grounded in the competencies gained through education, training, and experience.

Nursing and nursing assistance encompasses a spectrum of responsibilities, including treatment, nursing care, case management, and monitoring, all directed toward improving the health and quality of life for patients, their families, and the broader community. Nurses are also actively engaged in health promotion and patient education, contributing to holistic patient care. The expertise nurses bring to their roles is developed and acquired not only through a combination of education, training, and practical experience but also their innate drive for innovation and spontaneous insight-driven decision-making in varying critical junctures in nursing, demanding the same. The nurses, in turn, get accolades for their job, which motivates nurses and enhances the nursing experience.

The following statement by a patient further illustrates the patient’s ability to trust the expertise of the nurses, which counts for enhancing the patient outcomes as well as the nursing experience,

*Suppose I have some constipation problem. Nurses here take care of that. Taking care that I am taking certain medicines, even when I don’t want to take the medication, but they always insist. They give family-like attention. Very caring. (C-1-F-80).*

*Descriptive code: Compassionate Nursing Care.*

Nurses and nursing assistants approach their caregiving responsibilities with insight, knowledge, adaptability, and vigilance. Each patient case or situation is unique, and many are highly complex, requiring nurses to understand each case and develop patient-centered or personalized solutions. Therefore, the patients are satisfied with the services at SMOs.

This is exemplified by the statement of a patient receiving homecare services:

*I am satisfied with the homecare. My family and I feel relieved as the nurses have become more like a family to us. They take care of my recreational and spiritual needs. (C-1-F-80).*

*Descriptive code: Comprehensive Homecare with a Personal Touch.*

A statement from a nurse emphasizes why and how patients’ trust in nurses’ expertise enhances the nursing experience for her,

*I will provide good results and benefits to patients so that the patients can trust me and I am the face of the organization as they trust me. Their trust enhances my creativity through insightfulness and apt response to patient concerns in a way. (N-1-F-34).*

*Descriptive code: Building Trust, Delivering Excellence.*

Patient Trust and Support is the linchpin of building strong patient-nurse relationships firmly grounded in the competence, education, training, and experience of nurses and nursing assistants. Their ability to comprehend each unique case and spontaneously solve patient concerns with the application of insightfulness and ability to provide innovative personalized care adds an invaluable layer of trust in the homecare services. It leads to better patient outcomes and enhanced nursing experience.

## Discussion

In this study, nurses were found to actively employ advanced critical thinking and creativity in their daily tasks, offering both immediate and long-term solutions to patient care challenges while deviating from the traditional clinical focus. The self-management leadership theory highlights the significance of autonomy and creativity in nursing, aiming to enrich the nursing experience and improve care delivery [42]. Insightfulness emerges as a key construct that plays a pivotal role in helping nurses find profound meaning in their work, surpassing mere career aspirations. Additionally, self-managing teams in nursing play a crucial role in boosting job satisfaction through cultivating enhanced relationships, highlighting the paramount importance of meaningfulness in the nursing profession [3]. In SMOs, nurses find satisfaction and fulfillment by prioritizing compassionate caregiving, challenging the conventional career-centric approach. The autonomy provided by self-managing teams leads to satisfaction and brings joy to nurses [43]. Furthermore, the collective perception among nurses plays a pivotal role in shaping meaningful work experiences, challenging predominant individual-driven perspectives. The concept of shared meaning within the context of self-managing teams also emphasizes the collaborative nature of meaningfulness at work [44].

In SMOs the nurses are provided with valuable support and access to continuous learning opportunities, creating a synergistic effect that collectively enhances the overall experience for nurses in such organizational structures. The commitment to providing support and continuous learning is central to the success of self-managed teams. This commitment significantly contributes to elevated levels of job satisfaction among nurses [45–47]. Within the self-managing paradigm, nurses find support for their current roles and opportunities for further education and career advancement [48]. In addition to educational opportunities, SMOs celebrate the achievements of their nursing staff. This celebration goes beyond mere acknowledgment; it fosters a shared sense of purpose and fulfillment among the nurses. Recognizing accomplishments within the organizational framework contributes to creating a positive work culture [46], as highlighted by research findings. In this way, SMOs actively nurture an environment where nurses feel supported and engaged and experience a profound sense of accomplishment and purpose in their professional endeavors.

The study highlights the crucial importance of advanced critical thinking skills within nursing, particularly in the context of self-managed homecare organizations (SMOs). In the dynamic and multitasking environments of SMOs, nurses employ advanced critical thinking as a guiding principle, ensuring patient safety and the overall well-being of individuals under their care. This extends beyond traditional clinical boundaries, exploring diverse perspectives encompassing fairness, ethics, and evidence-based practice. The study underscores the foundational significance of critical thinking in nursing, emphasizing its pivotal role in delivering exceptional patient care [47]. Aligned with the philosophy of SMOs, which advocates for nurses to exceed assigned responsibilities [48], the study highlights nurses’ dedication to excellence by surpassing designated roles. Moreover, it emphasizes the indispensable nature of critical thinking in home care scenarios, particularly crucial for intensive care nurses [49]. This underscores the critical role of advanced thinking skills in navigating complex healthcare situations, ultimately ensuring optimal patient outcomes.

The study establishes a profound link between holistic critical thinking and success in terms of quality of care delivery, underscoring the pivotal role of considering fairness, ethics, and evidence-based practice in nursing [50]. The study highlights that heightened recognition and appreciation resulting from critical thinking significantly contribute to an enhanced nursing experience, fostering improved patient outcomes and service excellence [47].

The research outlines diverse strategies for optimizing the nursing experience, encompassing additional job support, group harmony, compact and specialized teams, role rotation, and nurturing critical thinking. Information and Communication Technology (ICT) solutions are crucial for job enrichment, streamlining task management, and communication within nursing teams. This aligns with existing studies emphasizing the positive impact of technology-based educational tools on nursing, enhancing healthcare functions and nurses’ capabilities [51]. The study discusses the utilization of Learning Management Systems (LMS) and microlearning, aligning with evolving trends in online training and recognizing the importance of accessible learning opportunities for nurses [52].

This research advocates for smaller team sizes in self-managed homecare organizations as one of the key constructs, citing their instrumental role in enhancing nurses’ job performance and creating a superior nursing experience. This approach fosters a patient-focused approach, minimizes patient interactions with multiple team members, and promotes efficient team communication, contributing to job enrichment and elevated job satisfaction [53, 54]. The study also underscores the positive impact of long-term team relationships on patient satisfaction and nursing experience, emphasizing the importance of continuity of care and familial bonds in the homecare setting [55]. Additionally, SMOs cultivate a proactive and entrepreneurial mindset among nursing professionals, fostering a culture of innovation and transformative change, ultimately contributing to improved patient care and organizational excellence [56]. This strategic focus on preserving a unique work culture through training enhances job satisfaction and contributes to a more fulfilling nursing experience. The SMOs also accept the natural departures of cultural misfits that, in turn, create a supportive work environment, reducing job insecurity, and promoting a respectful atmosphere, further enhancing the overall nursing experience [57].

Orientation training programs are integral in empowering nurses, playing a pivotal role in their professional development. These comprehensive initiatives encourage critical assessment and foster a culture of continuous improvement, contributing significantly to a more enriching nursing experience [58]. Employee fit assessment, achieved through immersive experiences, ensures alignment within the organization and contributes to an enhanced nursing experience. Transparent alignment evaluation emphasizes the importance of cultural fit in nursing organizations, enhancing overall cohesion and effectiveness while recognizing the delicate balance required for a rewarding nursing experience [57]. Acknowledging the principle of balancing autonomy and safeguards is crucial in SMOs, ensuring ethical practice and upholding high-quality care standards [56].

In SMOs, empowerment through education takes precedence in prioritizing nurses’ development. The pivotal role of education in nursing leadership is evident within SMOs, fostering a leadership-oriented environment [54, 59]. Moreover, patient trust within SMOs is highlighted as crucial, grounded in competencies, fairness, ethics, and evidence-based practice, aligning with studies emphasizing its direct impact on nursing outcomes [60]. The multifaceted impact on the nursing experience within SMOs involves leadership playing a central role in advocacy, positive change, and personal/professional growth, albeit with some literature potentially overlooking broader societal impacts from nursing leadership in SMOs [54, 61].

Patients’ trust and confidence in the professional skills of nurses and nursing assistants emerged as another critical factor for the success of SMOs. The emphasis on building beneficial patient-nurse relationships through trust and support reflects the broader significance of robust interpersonal connections in healthcare [62]. The portrayal of nurses providing family-like attention aligns with the documented importance of compassionate nursing care in fostering patient satisfaction [62]. Furthermore, the involvement of nurses in a spectrum of responsibilities at SMOs, including treatment, nursing care, case management, and monitoring, aligns seamlessly with the broader concept of holistic patient care [63]. The acknowledgment of the uniqueness of each patient case and the need for personalized solutions resonates with the patient-centered care approach, significantly contributing to overall patient satisfaction, as evidenced by positive feedback from patients receiving homecare services. The study underscores the fundamental importance of patient trust in enhancing the nursing experience and fostering creativity among nurses [62]. Trust is recognized as a cornerstone in relationships among stakeholders in homecare SMOs, influencing both patient outcomes and the job satisfaction of nurses, in turn enhancing the nursing experience [63, 64].

## Conclusion

We live in a world with unique problems that must be dealt with, and we are on the edge of massive transformations. The unconventional ways of management are necessary in India’s homecare sector to enhance the nursing experience, which is a driver for achieving patient satisfaction and better-performing homecare organizations. Self-management offers many benefits, as discussed in this research paper, and a few organizations are coming up with self-managed organizational structures and consider themselves successful. Despite the early fears about implementing the self-managed system in India, the data revealed that the studied homecare SMO successfully gained and retained the patient base during and post-COVID pandemic through better nursing experience. The studied SMO has also successfully gained the trust of nurses and retained them even during the COVID-19 pandemic when the nursing workforce was in denial of service due to fear of infection. The main reason behind the astounding success of SMOs in India is the unique approaches to nurses’ autonomy and satisfaction, enhancing their overall nursing experience. The interviews revealed various qualitative success enablers of SMOs in India that can be extended to other homecare organizations implementing or planning a self-management organizational structure in India to establish homecare as a prominent parallel healthcare alternative to the hospital and nursing home setups. This will ease the burden on existing healthcare setups.

Trust is a human’s fundamental instinct. Therefore, trusting the staff and care providers makes sense when it comes to holding responsibility not because of external control but with a spirit of serving people with a sense of one’s duty. The study of the given self-managed homecare organization revealed that SMOs could save a lot of unnecessary bills resulting from excessive bureaucracy characterized by hierarchical and complex management systems. The study showed that homecare sector SMOs in India are efficient, practical, and affordable, as suggested by patients in the interviews. The data indicates that protecting and expanding an SMO’s trust-based ecosystem, leading to a better nursing experience, is essential to effective service delivery. The nurses are innovative at SMOs, as creativity is always appreciated, facilitated, and inculcated in all the SMO members, which helps provide better homecare. The nurses experience meaningfulness, which motivates them to go beyond their job roles as essential in homecare service delivery. The nurses with better nursing experience have a holistic view of seeing patients with a sense of responsibility for them. The nurses, therefore, are enabled to do their job more empathetically, altruistically, and like a family, contributing to a more satisfactory nursing experience.

## Limitations

The limitations revealed from the data analysis in this research paper were that the nurses in India are conditioned to work in a bureaucratic set-up where there is always someone above them in the hierarchy to guide them and direct them on what to do, how much to do, and when to do. Gaining access to an appropriate pre-skilled workforce for SMOs is difficult in India. Therefore, the employees need to be trained by organizations implementing self-management to be deconditioned to work more autonomously like owners with a greater expanse of responsibilities, which is an additional financial commitment for SMOs in India.

### Supplementary Information

**Additional file 1.**

## Data Availability

Data is available with the authors as interview transcripts and can be shared with the journal anytime. The authors share no data with any data repository due to privacy concerns.

## Abbreviations

*SMOs*: Self-Managed Organizations
*MNCs*: Multi-National Companies
CGT: Constructivist Grounded Theory
*COVID-19*: Corona Virus Disease 2019
*ROI*: Return on Investment
*HR*: Human Resource
NP: Nursing Practitioner
*IT*: Information Technology
*CIO*: Chief Information Officer
*CEO*: Chief Executive Officer
*GNM*: General Nursing and Midwifery
*B.Sc. Nursing*: Bachelor of Science in Nursing
*EM*: Elham Malik (Author One)
*SS*: Shail Shankar (Author Two)
