# Supplementary material for: Empowering nurses: exploring self-managed organizations in Indian healthcare
Source: BMC Nurs. 2023 Dec 15;22:477. doi: 10.1186/s12912-023-01647-5 (PMC10722781; doi:10.1186/s12912-023-01647-5)
Supplement: Supplementary file 1 — Additional file 1. [file 12912_2023_1647_MOESM1_ESM.docx]

# Client Interview Questions

**Demographics**

Age?

Gender?

Year of joining the services?

Ailment/Need of care?

Family structure?

How do they get involved in your care delivery? Could you tell me something about the kind of support you have in care delivery from your family?

When did you first subscribe to Buurtzorg Homecare? Was the care delivery continued by you till now, or was it intermittent?

**Finances**

If possible, could you please tell me something about the payment structure at Buurtzorg? Is the fee charged per day/monthly/annual basis?

What is the average fee for services availed by you?

Could you compare the payment structure at Buurtzorg with your previous homecare organization?

Do you find home care delivery affordable?

Does the government provide for homecare expenses under the public healthcare system?

Could you tell me something about your satisfaction with the services provided at Buurtzorg versus the cost of services?

Do you find Homecare delivery affordable?

**Organizational Structure**

Do you feel involved in the decision-making process for care delivery?

Do you take support of your networks in care delivery besides care from Buurtzorg? If yes, how does it help you?

Are you able to invest trust in the caregivers? If yes, why? If not, why? What role does the organizational structure play in it?

Are there ways to share the best practices among caregivers? Please reflect.

Do you think that the way of care delivery by Buurtzorg is different from other providers? If yes, how?

What is the number of nurses who visit you per month? What do you think about the current arrangement of duties?

Have you taken any other homecare service before? Could you please compare Buurtzorg's experience with that in other organizations?

Could you please tell me about your experience at the previous homecare organization?

Could you please tell me about your homecare experience at Buurtzorg?

What changes would you suggest in Buurtzorg’s care-delivery system?

**Value System**

*Do you feel, in general, that the health care system follows a certain value system? Every organization has a certain value system, something that they value above everything in job delivery. (For example, organizations prefer trustworthiness, dependability, honesty, altruism, philanthropy, and independence; some are profit-driven, and others are value-driven……)*

What kind of value system do you think this system has/follows? Can you please compare it with your previous homecare organization (if any)?

Do you feel dependent on nurses? What do you think about it? Frustration or gratitude? What is the role of the value system of nurses in framing this opinion of yours? (below one’s are leading questions)

Do you think that nurses serve beyond their role? If yes, then how?

Do you find nurses well-behaved and agreeable-natured? If yes, how? If not, how?

Do you feel greater independence at Buurtzorg? If yes, how?

Do you feel more disciplined at self-care with Buurtzorg? If yes, how? If not, why?

Could you please tell me if you would like to get involved in the caretaking of those in need once you recover?

**Creativity and Performance Management**

Do you see any comparisons between the previous and the new organization in terms of creativity and performance?

What improvements in care delivery would you recommend?

What additional services would you like Buurtzorg to offer in the future that you currently feel are missing, if any? What services did your previous organization avail (if any)?

What is the level of ease with which you think Buurtzorg delivers care? How do you find the service delivery experience as compared to other providers?

Could you please tell me about any kind of physical activities you got involved in by Buurtzorg or their recommendation for better health and recovery, if any? How did they help you achieve better health outcomes if they positively affected your health?

Would you prefer a caretaking job as a nurse? If yes, how would you manage that job?

**Concept and purpose**

Do you think that Buurtzorg works towards making you independent in terms of care in the long term? If yes, how? If not, what do you think could be done for the same? Could you compare the independence strategy with your previous organization?

Did you ever have to approach management for discrepancies in service delivery? If yes, why? If yes, did you find a satisfactory solution? If yes, does it inculcate a tendency to trust in you? Could you compare the trust with the previous organization (if any)?

Do you think Buurtzorg serves its motto, “Humanity above bureaucracy? Reflect on the needs of paperwork required or the degree of intimations via phone calls or the internet required to get desired care delivery.

Please reflect on the rate of recovery at Buurtzorg. Could you compare it with the previous organization?

Would you recommend Buurtzorg to your acquaintances? If yes, why? If not, why?

**Coordination**

Did you face any unforeseen circumstances during care delivery where the nurse had to make an important decision? If yes, what was that experience, and how did the nurse handle it? Do you think other care providers would have dealt with it differently?

If you could tell me something about the coordination among the nurses, do you find a great deal of coordination among nurses? In unforeseen circumstances, how does the coordination among nurses play a role? Compare.

Are you requested to provide regular care delivery feedback at Buurtzorg? If yes, are the issues indicated resolved timely? What does it indicate about coordination in Buurtzorg, according to you?

**Information Flow**

Do you have access to needed information about homecare delivery?

Do you have access to the financial information of the company? Reflect on profit-making and sharing.

**Conflict Resolution and Dismissal**

Do you think that the nurses are trained in conflict management? Reflect?

How do nurses resolve conflicts among themselves and with patients, if any, during care delivery? Reflect. Do you have any precedent about this?

Was any of the nurses dismissed because of escalated unresolved conflict? If yes, reflect on the incident.

Are you satisfied with the current conflict resolution process? If not, reflect on the ideas you would like to be implemented for conflict management.

Are you satisfied with the conflict resolution mechanism at Buurtzorg?

Have you ever been in conflict with a nurse during care delivery? If yes, what was the reason for the conflict, and how was it resolved?

Could you compare the conflict resolution mechanism with your previous homecare provider (if any)?

# Nurse Interview Questions

**Demographics**

Age?

Gender?

Years of experience?

Years of working with Buurtzorg?

**Finances**

What are productivity or task requirements at Buurtzorg? Please reflect on the related payment structure.

What are the criteria for a salary hike?

Do you arrange resources, equipment, and impedimenta required for homecare delivery, or is the procurement done by Buurtzorg management? If you do, how do you arrange them?

Are you satisfied with the pay structure at Buurtzorg? Could you compare it with your previous employer?

Do you receive any fringe benefits like bonuses? Compare with the previous employer?

What are the criteria of compensation for job roles at Buurtzorg? Does it depend on seniority? Does it depend on any specific performance criteria?

**Organizational Structure**

Is it contractual employment at Buurtzorg? What is the renewal process?

The number of team members serving a patient?

Do you take help from informal support structures for care delivery, say, neighbors, relatives, and friends of clients? If yes, how do you find them helpful?

Do you think that being directed by the management is better or that being autonomous more conducive to your job delivery? How is it so?

What changes can Buurtzorg India make to ease care delivery and even improve the quality of care provided?

How can Buurtzorg improve the work environment for the nurses, according to you?

How do you think the trust in Buurtzorg can be fostered among the clients? Suggest ways through changes or improvement in organizational structure?

Did you witness team consensus and team role rotation at Buurtzorg? What do you think about team consensus and team role rotation at Buurtzorg?

How do you experience autonomy at Buurtzorg? How does organizational structure help in achieving autonomy?

Do you seek guidance for your job tasks at times? If yes, from whom?

Do you feel accountable for your job in the current organizational structure? How and why?

How many clients are there per nurse on average? How does this ratio help in better care delivery?

Have you worked in other homecare organization(s) before Buurtzorg? If yes, how would you compare the experience of working at Buurtzorg with other organizations?

Could you tell me about your experience of working with your previous employer? Could you compare the organizational structure of the previous employer organization?

Could you tell me about your experience working with Buurtzorg? Would you like to work with Buurtzorg in the future, too? If yes, why? If not, why?

**Value System**

*Do you feel, in general, that the health care system follows a certain value system? Every organization has a certain value system, something that they value above everything in job delivery. (For example, organizations prefer trustworthiness, dependability, honesty, altruism, philanthropy, and independence; some are profit-driven, and others are value-driven……)*

What drove you to the nursing profession? Reflect on your value system.

How do clients matter to you? Dimensions of patient viewed?

Do you take extra roles in care delivery? If yes, why and how?

Do you think your professional goals align with those of Buurtzorg? If yes, how? If not, why?

Do you feel trusted at Buurtzorg? How does your value system help in achieving trust at Buurtzorg?

Do you plan to work for Buurtzorg in the future, too? What retains you in the organization?

Do cultural values play a role in care delivery? How?

Do you employ indigenous wisdom in care delivery and, therefore, use regional resources? How?

Could you compare the value system at Buurtzorg with your previous employer organization?

**Creativity and Performance Management**

What are your expectations from the nursing profession? How do your expectations aid your creativity and performance, if they do aid in any way?

Do you customize services for patients? How do you accomplish it?

What are your educational qualifications? Does Buurtzorg provide learning opportunities? If yes, how? Are those opportunities self-guided?

Did working at Buurtzorg improve your skillset? If yes, how?

What is the rate of recovery of patients at Buurtzorg?

Do you feel trusted at Buurtzorg? If yes, how does it help in enhancing job performance? If not, why?

Please share your experience of working in Buurtzorg. Do you experience autonomy in task delivery? Does it improve your performance? If yes, how, according to you?

Is critical thinking encouraged? If yes, how does it help in better care delivery?

Did you feel burdened or comfortable with the outcomes of your decisions at the job? Were you wholly responsible, or was the responsibility well distributed among the team?

How do you practice awareness of what is needed in a particular situation during care delivery?

**Concept and purpose**

Do you feel like being in a leadership position at Buurtzorg? If yes, how? If not, why? How do your leadership abilities align with the organization’s purpose?

How do you see your patient? How do you take care of his emotional, spiritual, health, and mental needs?

Do you experience autonomy in task delivery? Does it improve your performance? If yes, how, according to you? How does it bring you nearer to the organizational concept and purpose?

Do you plan to establish a homecare organization in the future? If yes, how did Buurtzorg help awaken that drive?

Have you worked for other homecare organizations previously? Do you think Buurtzorg’s concept and purpose differ from other homecare organizations?

**Coordination**

Did you come across a situation where you had to manage an unforeseen circumstance during care delivery? If yes, how did you handle the situation? How did the coordination between nurses and your patient help manage that circumstance?

How well do you get along with your patients? Are they open to you telling their needs, likes, and dislikes?

Is there innate coordination among the nurses for care delivery? If yes, how? If not, how can it be inculcated? How would you compare the coordination at Buurtzorg with your previous employer organization?

**Job Titles/Job Descriptions and Compensation**

Are job titles or descriptions fixed, or are they granular? If granular, how does it help in extra-role behaviors?

If granular job titles are present, how does Buurtzorg compensate for the lack of job titles to keep employees motivated and productive?

Do nurses demand any kind of job titles from the organization’s management?

What are the criteria for compensation for jobs performed at Buurtzorg? Is it equal profit distribution or a salary system? Does it depend on specific job titles? Reflect.

Could you compare the job titles/descriptions and compensation with your previous employer organization?

**Information Flow**

Do you have access to needed information about homecare delivery?

Do you have access to the financial information of the company? Reflect on profit-making and sharing.

Could you compare the information flow with your previous employer's organization?

**Conflict resolution and dismissal**

What is the conflict resolution mechanism employed at Buurtzorg?

Are you trained in conflict management? How are conflicts resolved among nurses during care delivery?

Are you satisfied with the current conflict resolution process? If not, reflect on the ideas you would like to be implemented for conflict management.

What are the criteria for dismissal from service? Is there any such precedence please reflect.

Have you ever been in conflict with a patient? If yes, how was it resolved?

Have you ever been in conflict with a nurse? If yes, how was it resolved?

Could you compare the conflict resolution mechanism at your employer organization?

**Radical Decentralization of Authority**

People in self-managing organizations need not answer to a manager with broad discretion over their workdays, including assigning tasks, overseeing their completion, and setting their salaries and advancement prospects. How do you see radical decentralization in action at Buurtzorg?

**Formal System**

A self-managed organization (SMO) is a formal structure that specifies how power is distributed within the organization. For example, Morning Star, a Woodland, California-based agribusiness and food processing company, formalized its approach by outlining organizational principles for how employees should interact and a method for resolving workplace problems known as the "Gaining Agreement" procedure. Morning Star established a self-management institute as a think tank and educational institution to "define, refine, and promote the ideas and instruments of self-management in organizations. How is power distributed at Buurtzorg Edugreen?

**Pan-Organization**

In a self-managed organization, decentralization is not confined to the frontline staff or a specific team. The statutory regulations bind all employees, from the lowest-level workers to the highest-ranking executives. How is the pan-organization decentralization practiced at Buurtzorg Edugreen?

# Management Member Interview Questions

**Demographics**

Age?

Gender?

Career roles taken up in the past?

Years of experience?

Years of working with Buurtzorg India?

Could you please tell me about your view on hierarchy in organizations?

What are your views on flat organizational structures?

**Administration and Working**

National Standards for the homecare services followed at Buurtzorg Edugreen Neighbourhood Care India Pvt. Ltd.?

What kind of job is performed by the nurses? Do they give a sponge bath, change clothes, and do they need clean stools? Do they do domestic work, too? Are they specialized in a particular service or general nurses?

How is self-management implemented at Buurtzorg Edugreen Neighbourhood Care India Pvt. Ltd.?

Major people in administration and their roles at Buurtzorg Edugreen Neighbourhood Care India Pvt. Ltd.?

What is the average age group of patients at Buurtzorg Edugreen Neighbourhood Care India Pvt. Ltd.?

With regards to the teleconsultation in association with “To Get Well,” What is the current number of patients? Is it popular, and if so, what is the cutting-edge uniqueness compared to other organizations?

Basic infrastructure, tools, and equipment required at home as the minimum requirement for availing of nursing services? What do Medical Social Services and Counselling include?

With regards to companionship provisions at Buurtzorg India, what does light housekeeping work in elder care include? Does it involve running errands, fall prevention, and assisted exercise and walks, and daily vital checks?

With regards to remote monitoring services provided by Buurtzorg India, what job do care responders perform? (Visit by Care Responder Every Week)

How does the doctor visit every six months help in patients’ care delivery?

Please elucidate the “one logistic visit (max 2 hours) every month”, “conveyance to be paid as per actuals,” and “additional hours beyond 2 hours would be charged extra”, as mentioned on Buurtzorg India’s website.

In remote monitoring, what is the purpose of a logistic visit? Does the allotted time suffice for good care delivery?

What is the role of Buurtzorg Asia and Buurtzorg Nederland in Buurtzorg India’s functioning and administration?

**Finances**

Are the homecare services provided at Buurtzorg more affordable as compared to other homecare organizations?

How is the profit made at Buurtzorg India?

Any commitments for the nurses for earnings in terms of hours worked per week and income per hour?

Who manages finances at Buurtzorg, India? Each team versus a central authority that shares the profit according to any particular criteria? Do nurses give a share or the share of nurses is managed by the central authority?

Annual Turnover of Buurtzorg Edugreen Neighbourhood Care India Pvt. Ltd.? Share for the Buurtzorg Nederland? How is profit shared?

How does Buurtzorg Edugreen Neighbourhood Care India Pvt. Ltd. ensure the best possible patient service and that profitability is not the sole focus?

Are there any IT partners of Buurtzorg Edugreen Neighbourhood Care India Pvt. Ltd.? Please illuminate me on this.

Is there an average fee per hour with all the services grouped together, like at Buurtzorg Nederland, resulting in an average fee of around 57 Euros per hour?

At Buurtzorg Nederland, each team knows how much can be spent on renting offices and other expenses, and there is also accountability on how profits are invested, for example, for innovative projects, education, and training. Are there any such provisions at Buurtzorg Edugreen Neighbourhood Care India Pvt. Ltd.?

Which ones are the financing bodies for homecare in India, like in Nederland (two financing bodies: healthcare financing agencies and local municipalities)?

What are the cost/charges and package structure for palliative care?

What are the charges and package structures for physiotherapy?

The pattern of charging the conveyance charges for the logistic visits in remote monitoring? How much are the additional charges as mentioned on the website?

**Organizational Structure**

Do you see Buurtzorg as a living entity with its own evolutionary purpose? If yes, how does Buurtzorg’s organizational structure serve this purpose?

What is your current experience of running Buurtzorg in India? Do you see any impending changes required in Buurtzorg’s organizational structure?

At Buurtzorg Nederland, each team knows how much can be spent on renting offices and other expenses, and there is also accountability on how profits are invested, for example, for innovative projects, education, and training. Are there any such provisions in Buurtzorg Edugreen Neighbourhood Care India’s organizational structure?

How does Buurtzorg Edugreen Neighbourhood Care India Pvt. Ltd. ensure the best possible patient service and that profitability is not the sole focus? How does Buurtzorg’s organizational structure help to achieve this?

Is the care holistic or fragmented? Who gives Injections? Who gives medications?

What are the working hours per nurse? Is it on a monthly or daily basis?

Are there self-managed teams? If there, then how many members are there in each team? Are the teams organized community or city-wise?

What is the formal organizational Structure at Buurtzorg Edugreen Neighbourhood Care India Pvt Ltd?

Are the informal networks of patients brought into use? If yes, how?

What is the impact of the current organizational structure on people’s behavior in Buurtzorg Edugreen Neighbourhood Care India Pvt. Ltd. India? (How are power balance, excessive bureaucracy, and inefficiency coped with?)

How do you see Buurtzorg as compared to other organizations in terms of organizational structure, working, client focus, employee care, and motivation?

**Value System**

Is Buurtzorg holistic in approach? If yes, how does it take care of all its stakeholders? Does the value system of stakeholders play a role in it? If yes, how?

Is Buurtzorg a revolutionary organization? If yes, how? Where do you see Buurtzorg Nederland going in terms of being a revolutionary organization in the homecare sector? How does Buurtzorg’s value system play a role in this?

How does the Indian value system affect homecare in India? Elucidate.

How do you see Buurtzorg’s value system as compared to the other organizations?

**Creativity and Performance Management**

How often are the executive team meetings conducted? How do meetings serve the creativity and performance enhancement at Buurtzorg? Compare

What strategies do you recommend for better care delivery by Buurtzorg in the future?

Necessary qualification of nurses and their educational opportunities at Buurtzorg Edugreen Neighbourhood Care India Pvt. Ltd.? Do they have a budget for education and training? How do the current criteria serve the creativity and performance enhancement of nurses?

What are the methods of measurement of client satisfaction at Buurtzorg Edugreen Neighbourhood Care India Pvt. Ltd.? Compare.

What are the methods of measurement of employee satisfaction at Buurtzorg Edugreen Neighbourhood Care India Pvt. Ltd.? Compare.

**Concept and purpose**

Do you see Buurtzorg as a living entity with its own evolutionary purpose? How does the conception of Buurtzorg play its role in it?

Is Buurtzorg’s approach a long-term approach with a focus on the present? Please elucidate.

Do you plan the expansion of the organization to other cities in India to serve the organization’s concept and purpose? Reflect.

What are the methods of measurement of client satisfaction at Buurtzorg Edugreen Neighbourhood Care India Pvt. Ltd.? How does it serve the organization’s concept and purpose?

What are the methods of measurement of employee satisfaction at Buurtzorg Edugreen Neighbourhood Care India Pvt. Ltd.? How does it serve the organization’s concept and purpose?

**Coordination**

What are the density of clients in the area of operation, the resources available in the area necessary for Homecare, and the cultural setting? How does coordination among nurses-nurses, patient-nurses, and management-nurses help in achieving the utilization of regional resources and incorporation of cultural values?

How is nurse-nurse coordination achieved at Buurtzorg India?

How is the client-nurse coordination achieved at Buurtzorg India?

How is the management-nurse coordination achieved at Buurtzorg India?

Are frequent meetings required to achieve coordination at Buurtzorg, India?

How would you compare coordination at Buurtzorg with other homecare delivery organizations?

**Job Titles/Job Descriptions and Compensation**

Are job titles or descriptions fixed, or are they granular? If granular, how does it help in extra-role behaviors?

What kind of job is performed by the nurses? Do they give a sponge bath, change clothes, and do they need clean stool? Do they do domestic work, too? Are they specialized in a particular service or general nurses? How do current job titles help in the easy delivery of services?

Are there demands from patients for any kind of additional services not provided at Buurtzorg presently? If yes, what are they? What will be the job descriptions given to such employees?

Are the nurses compensated based on their current job titles, seniority, and performance? Elucidate.

**Information Flow**

Is real-time information available to the nurses? Are financial details disclosed to the stakeholders transparently? Compare with the other homecare organizations?

Are there any virtual platforms for effective schedule planning for the nurses, a forum for sharing experiences, developing innovative solutions to problems through joint effort, sharing knowledge, client registration, treatment times, and communication history?

Nurses need access to all information required for autonomous functioning while working locally in offices, on the road, and in patients’ homes in the case of self-managed teams. Is there an ICT company for Buurtzorg Edugreen Neighbourhood Care India Pvt. Ltd. to accomplish such autonomy? If not, how is it accomplished?

**Conflict Resolution and Dismissal**

What is the conflict resolution mechanism at Buurtzorg, India? Is there any role for regional coaches at Buurtzorg Edugreen Neighbourhood Care India Pvt. Ltd. for the same?

Are the clients satisfied with the current client-nurse conflict resolution mechanism? If yes, how? If not, why?

Are the nurses satisfied with the current nurse-nurse conflict resolution mechanism? If yes, how? If not, why?

Are the nurses satisfied with the current client-nurse conflict resolution mechanism? If yes, how? If not, why?

Do you think changes are needed in the current conflict resolution mechanism as per the Indian context? If yes, what are they?

What are the criteria for the dismissal of nurses during the conflict resolution process? Are there any precedents?

Could you please compare the conflict-resolution mechanism at Buurtzorg with the conflict-resolution mechanism in other homecare organizations?

**Radical Decentralization of Authority**

People in self-managing organizations need not answer to a manager with broad discretion over their workdays, including assigning tasks, overseeing their completion, and setting their salaries and advancement prospects. How do you see radical decentralization in action at Buurtzorg?

**Formal System**

A self-managed organization (SMO) is a formal structure that specifies how power is distributed within the organization. For example, Morning Star, a Woodland, California-based agribusiness and food processing company, formalized its approach by outlining organizational principles for how employees should interact and a method for resolving workplace problems known as the "Gaining Agreement" procedure. Morning Star established a self-management institute as a think tank and educational institution to "define, refine, and promote the ideas and instruments of self-management in organizations. How is power distributed at Buurtzorg Edugreen?

**Pan-Organization**

In a self-managed organization, decentralization is not confined to the frontline staff or a specific team. The statutory regulations bind all employees, from the lowest-level workers to the highest-ranking executives. How is the pan-organization decentralization practiced at Buurtzorg Edugreen?

**Self-management in India**

Why must self-management be given a chance in the Indian context?

What checks and balances are applied to implement self-management in the Indian context?

What are the outcomes of these checks and balances for Buurtzorg Edugreen?

Do nurses know exactly what they are trying to accomplish and what it means to be a part of a self-managed organization?

What is meant by self-management as it happens in action in the Indian context?

What are the aberrations witnessed in implementing self-management in organizations in India, and what are their causes?

What processes in organizational settings/ work settings, as accustomed to the Indian context, mean self-management?

What can be done to improve self-management in organizations in India?

What government policy changes are needed to implement self-management in India successfully?
